# Genetically predicted allergic rhinitis causally increases the risk of erectile dysfunction 

**DOI:** 10.3389/fgene.2024.1423357

**Published:** 2024-07-24

**Authors:** Peng Li, Zhaotun Meng, Liqiang Lin, Zhipeng Chen, Huaiqing Lv

**Affiliations:** ^1^ School of Clinical Medicine, Shandong Second Medical University, Weifang, China; ^2^ Department of Otorhinolaryngology, Linyi People’s Hospital, Linyi, China

**Keywords:** allergic rhinitis, erectile dysfunction, Mendelian randomization, causal estimates, statistic method

## Abstract

**Objective:**

Evidence shows that allergic rhinitis (AR) may increase the risk of erectile dysfunction (ED). This study aims to investigate whether there is a causal relationship between AAR and ED by Mendelian randomization (MR) analysis.

**Methods:**

We performed a two-sample MR analysis using genome-wide association studies (GWAS) summary data. Single nucleotide polymorphisms (SNPs) associated with AR and ED were obtained from the GWAS database. The MR analysis primarily employed the inverse variance weighted (IVW), MR Egger, and weighted median (WM) methods. We assessed pleiotropy using the MR-PRESSO global test and MR-Egger regression. Cochran’s Q test was used to evaluate heterogeneity, and a leave-one-out analysis was performed to verify the robustness and reliability of the results.

**Results:**

The IVW analysis demonstrated a positive association between genetic susceptibility to AR and an elevated relative risk of ED (IVW OR = 1.40, *p* = 0.01, 95% CI 1.08–1.80). The results obtained from MR-Egger regression and WM methods exhibited a consistent trend with the results of the IVW method. Sensitivity analyses showed no evidence of heterogeneity nor horizontal pleiotropy. The leave-one-out analysis showed that the findings remained robust and were unaffected by any instrumental variables.

**Conclusion:**

This study presents genetic evidence that indicates a causal association between AR and ED.

## Introduction

Erectile dysfunction (ED), defined as the consistent or recurrent inability to achieve and maintain an erection sufficient for satisfactory sexual intercourse, is a commonly encountered disorder in men, significantly impacting both individual wellbeing and the relationships of couples (McCabe et al., 2016; Krzastek et al., 2019). The worldwide prevalence of ED ranges from 14% to 48%, in the United States alone, at least 12 million men aged 40 to 79 are affected by ED (Sangiorgi et al., 2021). According to Feldman et al.’s epidemiological survey, conducted as part of the Massachusetts Male Aging Study, the prevalence of minimal, moderate, and severe ED among men aged 40 to 70 was 52% (Feldman et al., 1994). According to projections, it is estimated that there will be 322 million cases of ED by 2025 (Ayta et al., 1999). Therefore, identifying ED risk factors and assessing individuals who might benefit from proactive prevention or early intervention are crucial.

Factors such as smoking, alcohol consumption, depression, anxiety, sleep disorders, obstructive sleep apnea syndrome, cardiovascular diseases, chronic obstructive pulmonary disease (COPD), asthma, and diabetes, have been identified as associated with the onset of ED (Chou et al., 2011; Nguyen et al., 2017; Li et al., 2023). While allergic rhinitis (AR) has been reported to be related to ED, the literature on this association is scarce (Chiang et al., 2024). AR, along with COPD and asthma, belongs to the category of chronic inflammatory airway diseases. This condition, commonly affecting the nasal mucosa and often overlooked, is characterized by symptoms such as itching, sneezing, runny nose, and nasal congestion (Greiner et al., 2011). AR is a globally recognized concern, and its incidence has sharply increased in recent years. In the United States, 14% of adults, or one in every seven, are diagnosed with AR by a physician (Meltzer et al., 2009). In Denmark, the prevalence of AR in adults has gradually increased from 19% to 32% over the past 3 decades (Leth-Moller et al., 2020). A retrospective matched controlled cohort study, comprising 128,118 participants (64,059 AR patients and 64,059 controls), revealed that during a follow-up period of 5.82 years, the prevalence of ED was higher among AR patients compared to the control group (95% CI, 1.24–1.52; *p* < 0.001) (Su et al., 2013). Based on the data collected from the self-administered questionnaires, Kirmaz et al. concluded that patients treated for allergic rhinoconjunctivitis demonstrated significantly higher International Index of Erectile Function scores compared to those experiencing allergic rhinoconjunctivitis symptoms concurrently (*p* = 0.01) (Kirmaz et al., 2005). Benninger et al.’s research consolidated questionnaire survey findings from multiple cohorts, culminating in the conclusion that AR negatively affects sexual function (Benninger and Benninger, 2009). However, the further influence of AR on ED is rarely investigated, with scant literature available on the subject.

The Mendelian randomization (MR) analysis is a method for determining the causality between exposure and outcome by using genetic variations as instrumental variables (IVs) (Emdin et al., 2017). The basis of MR analysis lies in the random allocation of alleles for these genetic variants during conception, which supports inferring causality between exposure factors and outcomes. This approach is unaffected by the reverse causality and reduces the influence of confounding and bias in the study (Evans and Davey Smith, 2015; Emdin et al., 2017). Compared to traditional observational studies, MR offers advantages such as cost savings, time efficiency, high feasibility, and reduced susceptibility to confounding factors. This method has not been applied before to explore the relationship between ED risk and AR, though. In this study, we used the two-sample MR method approach to investigate the causal relationship between AR and ED.

## Methods

### Study design and ethics statement

**FIGURE 1:**
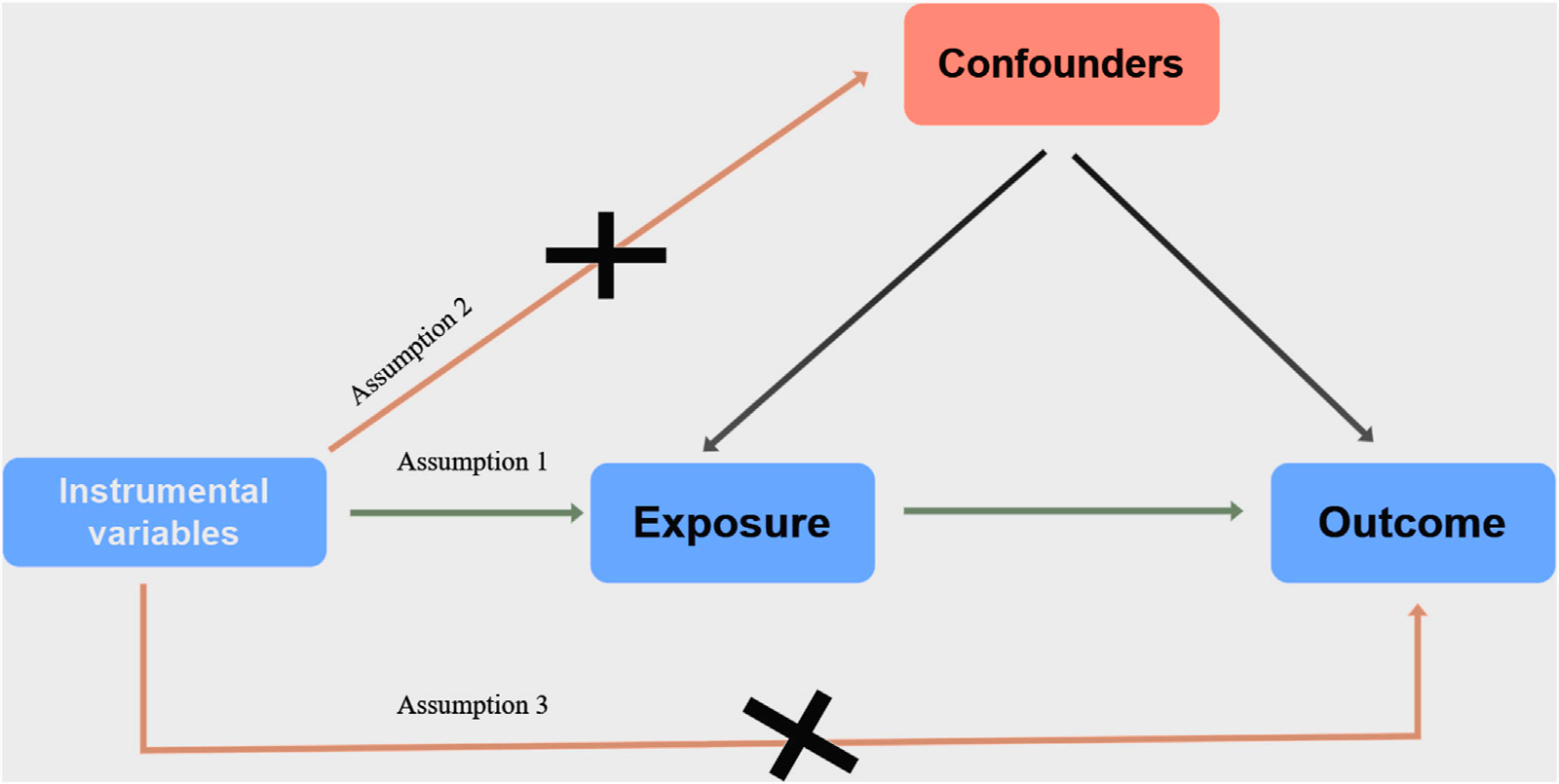
Diagram of Mendelian randomization (MR) study design.

In this study, we employed a two-sample MR analysis to investigate the causal relationship between AR and ED. The foundation of MR analysis relies on three assumptions. Firstly, there should be a strong correlation between the exposure of interest and IVs, represented here as single nucleotide polymorphisms (SNPs). Secondly, there should be no confounding factors associated with the IVs. Finally, the IVs should only affect the outcomes through the exposure of interest (Lawlor et al., 2008). All the data utilized in the MR analysis were extracted from the public genome-wide association studies (GWAS) database (https://gwas.mrcieu.ac.uk). The combined dataset comprised information from previously conducted studies, all of which had obtained approval from their respective institutional review boards. Therefore, ethical approval was not required for this study. Figure 1 shows an overview of the study’s design.

### Data sources

In our study, we designated AR as the exposure and ED as the outcome. Data on AR and ED were sourced from publicly available large-scale GWAS datasets, accessible for download from the MRC IEU Open GWAS dataset. The summary statistics for AR exposure were obtained from GWAS ID: ebi-a-GCST90086042, which comprised 13,936 cases and 42,701 controls. To ensure no overlap between exposed populations and those with outcomes, the outcome dataset was sourced from FinnGen (finn-b-ERECTILE_DYSFUNCTION), containing 1,154 cases and 94,024 controls. All participants were of European ancestry. The summary statistics for two GWAS datasets were provided by the IEU Open GWAS Database.

### Instrument variable selection

In this study, we identified SNPs significantly associated with AR (*p* < 5 × 10^−6^) from GWAS summary data. To ensure independence among instrumental factors, we employed the clumping technique for each exposure, setting the threshold at *r* ^2^ = 0.001 and a window size of 10,000 kb to filter out SNPs in significant linkage disequilibrium. During this process, we excluded 1 palindromic SNP (rs2477914). We then screened for genetic variants associated with potential confounding factors in the Phenoscanner database (http://www.phenoscanner.medschl.cam.ac.uk/). Additionally, we assessed the strength of the IVs using the F-statistic, calculated as F = beta^2^/se^2^ (Feng et al., 2022). Generally, an F statistic <10 indicates weak IVs. We retained 16 SNPs as IVs for subsequent MR analysis. Detailed information on the IVs is provided in Supplementary Table S1.

### Statistical analyses

To assess the causal relationship between AR and ED, we employed primarily the Weighted Median (WM), MR-Egger, and Inverse Variance Weighted (IVW) methods. Among these, IVW emerged as the most robust (Bowden et al., 2015). The IVW analysis involved the use of the IVW meta-analysis approach, which estimated MR by computing the Wald ratio for each SNP. This method assumes a zero intercept and requires all SNPs to be valid IVs. IVW is preferred in MR analysis when no directional pleiotropy is observed among SNPs, as it can provide reliable estimates (Pierce and Burgess, 2013; Burgess et al., 2015). In the presence of evidence for pleiotropy, MR-Egger regression is favored. The condition for estimating causal effects using WM is that at least half of the SNPs are valid IVs (Bowden et al., 2016). We assessed the pleiotropy of IVs using MR-Egger regression and MR-PRESSO global test. Cochran’s Q statistic was employed to evaluate heterogeneity. Leave-one-out analysis was conducted to identify any individual SNP that might introduce bias affecting the overall causal effect. The association between AR and ED risk was presented using Odds Ratios (ORs) and their corresponding 95% Confidence Intervals (CIs).

In this study, all statistical analysis was performed using R software (version 4.2.2, www.r-project.org) through the TwoSampleMR (0.5.7) package and MRPRESSO (1.0). A *p*-value <0.05 was deemed significant.

## Results

### Causal effects of genetically predicted AR on ED

**FIGURE 2:**
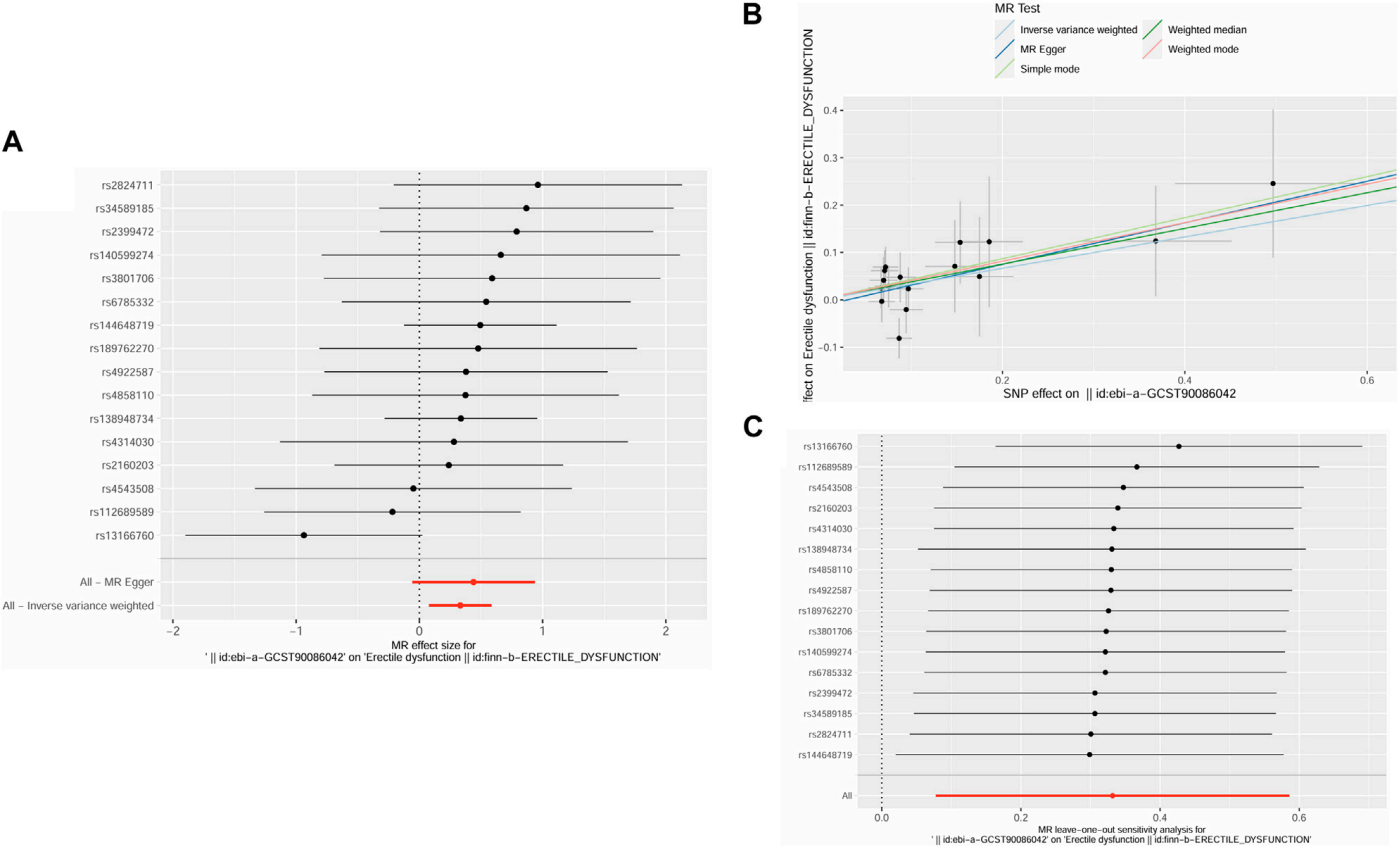
**(A)** Forest plot for the causality of each SNP on erectile dysfunction risk. **(B)** Scatter plot for the causality of AR on ED risk. **(C)** The leave-one-out plot presents how the causal estimates for the effect of RA on ED was influenced by the removal of a single variant.

The F-statistics for these IVs (19.58–38.36) all exceeded 10, indicating that none were weak IVs. IVW analysis revealed a statistically significant causal relationship between AR and the risk of ED (OR = 1.39, *p* = 0.01, 95% CI = 1.08–1.80) (Table 1). A similar trend was observed in the WM method (OR = 1.46, *p* = 0.04, 95% CI = 1.02–2.08) (Table 1). The results were visually represented through the forest plot (Figure 2A) and scatter plot (Figure 2B). The forest plot displayed effect estimates and their corresponding confidence intervals for each SNP, offering a comprehensive view of the data. Meanwhile, the scatter plot showed an increase in SNP effects on ED with increasing SNP effects on AR. Furthermore, both the MR-PRESSO global test (Rssobs = 13.05, *p* = 0.79) and MR-Egger test (Intercept = −0.01, *p* = 0.63) did not detect pleiotropy, suggesting the absence of pleiotropy. Results from Cochran’s Q test (P_MR-Egger_ = 0.67, P_IVW_ = 0.7) indicated no heterogeneity (Table 2). Leave-one-out analysis (Figure 2C) revealed that individual SNPs had no significant impact on causal inference, indicating the robustness and reliability of the MR results.

**TABLE 1:** Mendelian randomization estimation of the causal effect between AR and ED.

Exposure outcome	MR methods	N SNP	Beta	SE	*p*-value	OR	95% CI	
AR ED	IVW	16	0.33	0.13	0.01	1.39	1.08	1.80
	MR-Egger	16	0.44	0.25	0.11	1.55	0.94	2.55
	WM	16	0.38	0.18	0.04	1.46	1.02	2.08
ED AR	IVW	7	0.01	0.02	0.65	1.01	0.97	1.05
	MR-Egger	7	0.01	0.04	0.79	1.01	0.93	1.10
	WM	7	0.03	0.03	0.32	1.03	0.97	1.08

Abbreviations: AR, allergic rhinitis; IVW, inverse variance weighted; WM, weighted median; SE, standard error; OR, odds ratio; CI, confidence interval; The criterion for determining statistical significance was a *p*-value < 0.05.

**TABLE 2:** Sensitivity analyses of MR.

Heterogeneity				Pleiotropy			
MR-Egger		IVW		MR-PRESSO global outlier test		MR-Egger regression	
Q statistic	*p*‐value	Q statistic	*p*‐value	Rssobs	*p*‐value	Intercept	*p*‐value
11.23	0.67	11.48	0.72	13.05	0.79	−0.01	0.63

IVW, inverse variance weighted; The criterion for determining statistical significance was a *p*-value <0.05.

### Causal effects of genetically predicted ED on AR

We utilized the same approach as the forward MR analysis to conduct reverse MR, identifying SNPs for this purpose. A total of 11 SNPs were selected as IVs for exposure (*p* < 5 × 10^−6^, *r* ^2^ = 0.001, kb = 10,000). The F-statistics for these IVs (20.87–27.36) all exceeded 10, indicating none were weak IVs. Subsequently, we excluded four palindromic SNPs (rs3133073, rs67376322, rs6936413, rs78093953) from the IVs, leaving 7 SNPs for the MR analysis (Supplementary Table S2). The results obtained using the IVW method did not provide statistical support for the hypothesis that ED could increase the incidence of AR (OR = 1.01, 95% CI = 0.97–1.05, *p* = 0.65) (Table 1). Similarly, consistent findings were observed in the MR-Egger (OR = 1.01, 95% CI = 0.93–1.10, *p* = 0.79) and WM (OR = 1.03, 95% CI = 0.97–1.08, *p* = 0.32) (Table 1). Furthermore, Cochran’s Q statistic revealed no evidence of heterogeneity in causal inferences (P_IVW_ = 0.58, P_MR-Egger_ = 0.45). The MR-Egger intercept test results suggested the absence of horizontal pleiotropy (Intercept = −0.001, *p* = 0.95. Additionally, the leave-one-out analysis did not identify any outliers (Supplementary Figure S1).

## Discussion

Exploring the relationship between AR and ED is challenging due to the lack of rigorously controlled clinical trials and longitudinal prospective studies. In this study, we utilized a two-sample MR analysis to investigate the causal relationship between AR and the risk of ED. To our knowledge, this study is the first to explore this causal relationship using a large-scale GWAS dataset in MR analysis. Additionally, reverse MR analysis was conducted to validate our findings.

AR is a globally recognized concern, with a recent surge in incidence observed across different regions. For instance, in the United States, approximately 14% of adults receive a physician-diagnosed AR (Meltzer et al., 2009), while in Denmark, the prevalence of AR among adults has steadily climbed from 19% to 32% over the last 3 decades (Leth-Moller et al., 2020). Similarly, China has witnessed a 6.5% increase in the standardized prevalence of adult AR over the past 6 years (Zhang et al., 2021). In 2013, A retrospective matched controlled cohort study conducted in Taiwan, China, involving 128,118 participants (64,059 AR patients and 64,059 controls), found that AR patients had a higher likelihood of developing ED (log-rank test, *p* < 0.001), After adjusting for confounding variables through Cox regression, the incidence rate of ED in AR patients was 1.37 times higher than in the control group (95% CI, 1.24–1.52; *p* < 0.001), moreover, the risk of ED was further elevated in AR patients who had more frequent clinical visits and those requiring medication for over 4 weeks (Su et al., 2013). In 2005, Kirmaz et al. conducted a study based on self-administered questionnaires, which revealed that patients receiving treatment for allergic rhinoconjunctivitis had significantly higher scores on the International Index of Erectile Function compared to those experiencing symptoms concurrently (*p* = 0.01) (Kirmaz et al., 2005). Additionally, Benninger et al.’s research, which synthesized questionnaire survey findings from multiple cohorts, concluded that AR has a detrimental impact on sexual function (Benninger and Benninger, 2009).

Li et al. conducted an MR study, revealing that genetic predispositions to coronary artery disease and heart failure are associated with an increased risk of ED (Li et al., 2023). In a study by Matheson et al., which analyzed a cohort of 9,272 middle-aged and elderly individuals, AR was identified as a risk factor for incident stroke (Matheson et al., 2008). AR is not merely a localized ailment, its induced inflammation may have systemic implications (Borish, 2003). Research indicates that inflammatory mediators and cells associated with AR, such as leukotrienes, immunoglobulin E (IgE), and mast cells, participate in the process of atherosclerosis, which correlates with the occurrence of ED (Radmark, 2003; Schwartz and Kloner, 2011; Theoharides et al., 2011; Zhang et al., 2020). Leukotrienes may promote atherosclerosis by enhancing leukocyte chemotaxis, vascular inflammation, increased vascular permeability, and subsequent tissue/matrix alterations (Riccioni et al., 2009; Poeckel and Funk, 2010). IgE can modulate the expression of the MSRN gene controlled by IFN-γ, influencing macrophage polarization and foam cell formation in the progression of atherosclerosis (Zhang et al., 2020). Mast cells accumulate in the intima and adventitia of human arteries during the progression of atherosclerotic plaques, releasing vasoactive and angiogenic compounds, as well as pro-inflammatory mediators such as arachidonic acid metabolites, histamine, cytokines/chemokines, platelet-activating factor (PAF), and proteases enzyme (Spinas et al., 2014). Braunstahl et al. observed an increase in eosinophil infiltration in nasal and bronchial tissues, along with elevated expression of intercellular adhesion molecule-1 (ICAM-1), vascular cell adhesion molecule-1 (VCAM-1), and E-selectin, following 24 h of nasal allergen provocation in AR patients (Braunstahl et al., 2001). Cell adhesion molecules facilitate the adherence of circulating leukocytes and other blood cells to the activated vascular endothelium at inflammatory sites, which is also implicated in the pathogenesis of atherosclerosis (Hansson, 2005). Interestingly, elevated levels of circulating adhesion molecules were observed in ED patients without cardiovascular risk factors, further highlighting the potential association between AR and ED (Bocchio et al., 2004). Additionally, nasal congestion or runny nose during acute AR episodes may diminish libido in patients, thereby promoting the onset of ED (Rondon et al., 2012). These findings offer novel insights into the relationship between AR and ED.

In this study, we utilized a two-sample MR analysis to investigate the causal relationship between AR and ED. The MR principle ensures minimal bias from confounding factors and eliminates the possibility of reverse causation, providing stronger support for causal inference compared to observational studies. Additionally, as all participants were of European descent, potential biases from population stratification were minimized.

However, the study does have limitations. Firstly, the dataset only included individuals of European descent, limiting the generalizability of the findings to other ethnic groups. Secondly, the study did not differentiate between subtypes of ED, such as non-vascular or vascular ED. Future research could focus on analyzing these subgroups to provide a more nuanced understanding of ED. Thirdly, the study’s results only reflect the lifelong impact of AR on ED, leaving the short-term effects unclear. Prospective randomized controlled trials in the future could offer more comprehensive insights and validate our conclusions.

## Conclusion

This study unveils that genetically predicted AR might heighten the risk of ED.

## Data Availability

The original contributions presented in the study are included in the article/Supplementary Material, further inquiries can be directed to the corresponding author.
