# Supplementary figures and images for: Genetically predicted allergic rhinitis causally increases the risk of erectile dysfunction
Source: Front Genet. 2024 Jul 24;15:1423357. doi: 10.3389/fgene.2024.1423357 (PMC11303240; doi:10.3389/fgene.2024.1423357)

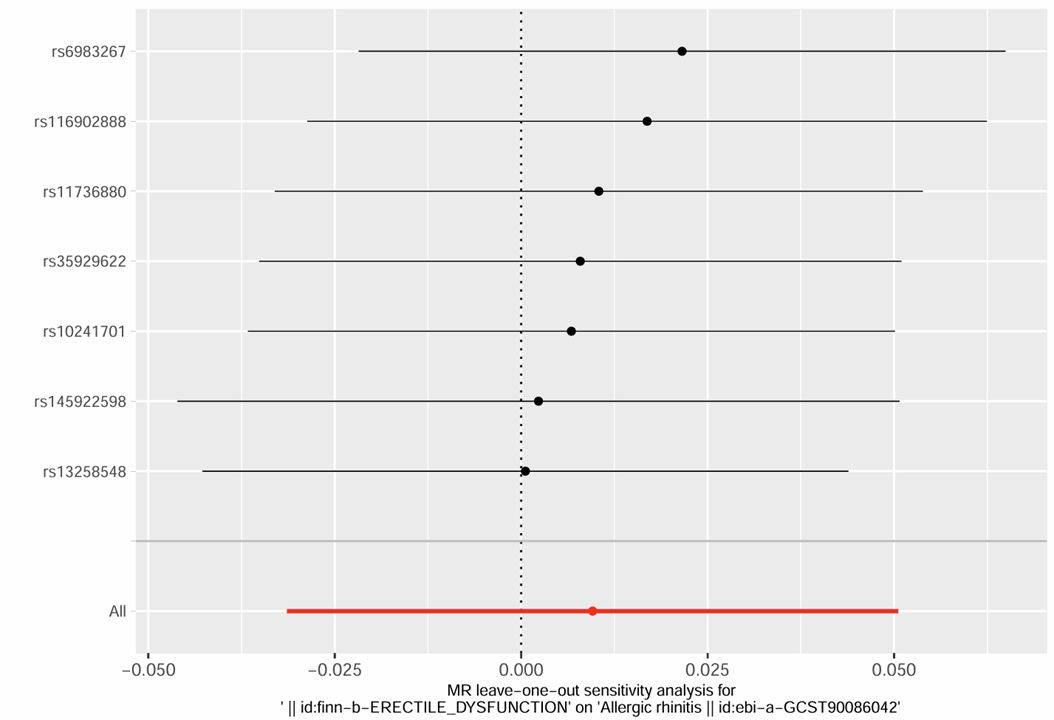

Supplement: Supplementary file 1 [file DataSheet1.ZIP › Supplementary Material Figure 1.jpg]
